# Supplementary material for: Metabolomic and gut microbiome profiles across the spectrum of community-based COVID and non-COVID disease
Source: Sci Rep. 2023 Jun 27;13:10407. doi: 10.1038/s41598-023-34598-7 (PMC10300098; doi:10.1038/s41598-023-34598-7)

Metabolomic and gut microbiome profiles across the spectrum of community-based COVID and non-COVID disease.

Authors:

Marc F. Österdahl[1]* & Ronan Whiston[1]*, Carole H. Sudre[1], Francesco Asnicar[2], Nathan J. Cheetham[1], Aitor Blanco Miguez[2], Vicky Bowyer[1], Michela Antonelli[1], Olivia Snell[1], Liane dos Santos Canas[1], Christina Hu[3], Jonathan Wolf[3], Cristina Menni[1], Michael Malim[1], Deborah Hart[1], Tim Spector[1], Sarah Berry[1], Nicola Segata[2], Katie Doores[1], Sebastien Ourselin[1], Emma L Duncan[1], Claire J Steves[1]

1) King's College London, London, United Kingdom

2) University of Trento, Trento, Italy

3) ZOE Global Ltd, London, United Kingdom

Corresponding Author: Marc F. Österdahl: marc.osterdahl@kcl.ac.uk

*Marc F Österdahl & Ronan Whiston should be identified as joint first authors.

Supplementary Methods

DNA Extraction

Briefly, 1g faecal sample was mixed with 5-6 ml of distilled water and 2 Core 5 mm glass beads. Tubes were put in Spex Grinder for 10 sec at 800. Contents settled for 12 mins, supernatant transferred to Core beat tube and centrifuged for 10 min at 15,000 g. Supernatant was removed and 400µl Core Clarifying solution was added. Bead beating of samples in Spex Grinder for 5 minutes at a rate of 1000. Samples were centrifuged for 3 minutes at 15,000 g. Proteinase K (10µl) was added to the deep well plate. 200µl of each sample was added to each well and mixed thoroughly. Finally, 720µl of Lysis/Bind Master Mix was added to each well. Plates were then run using King-Fisher Flex, as per manufacturer's instructions. Elution was in 100µl of MagMax Core elution buffer. gDNA quantity was estimated using Qubit.

Supplementary Figures

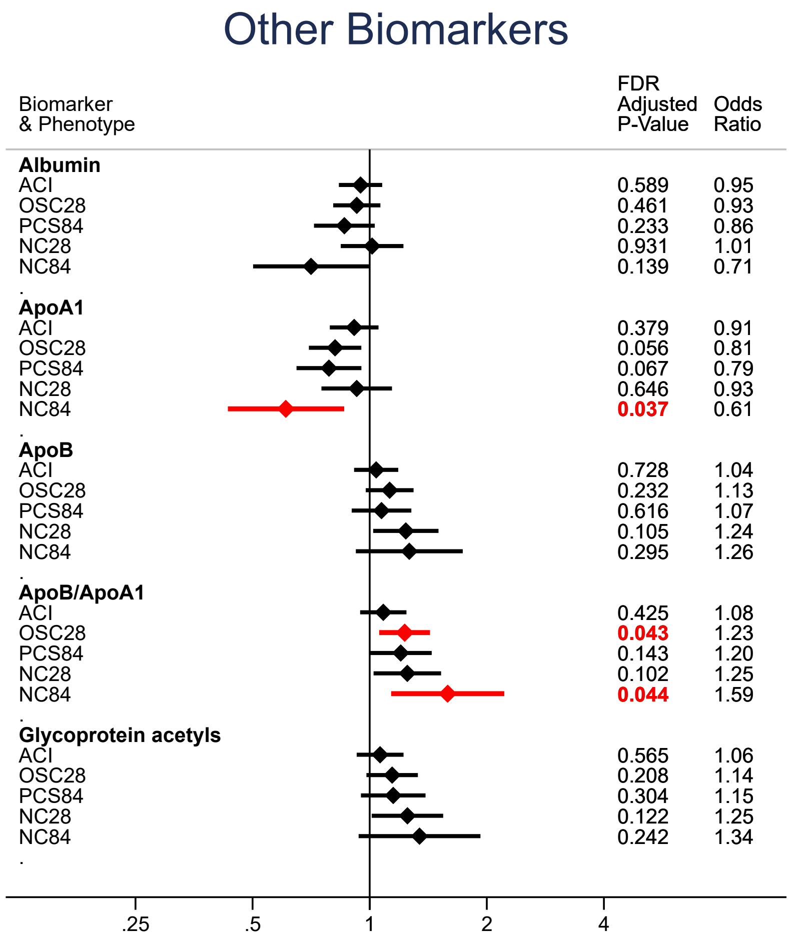

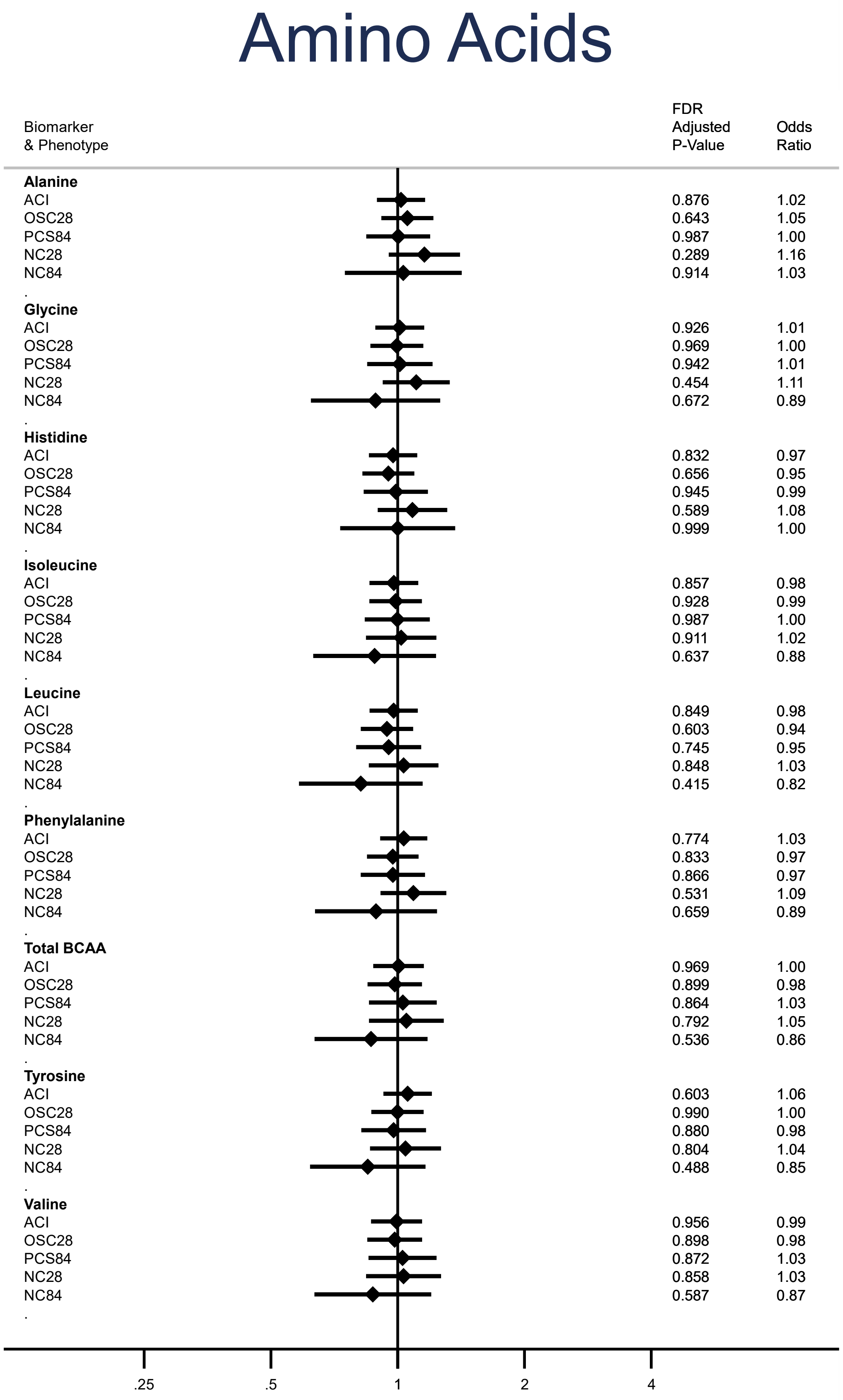

Supplementary Figure 1: Clinically validated amino acids and other clinically validated biomarkers.

Relative risk ratio for each illness phenotype, per 1-SD increase in biomarker.
Red indicates p≤0.05 after FDR correction
Reference group (OR 1.0): Asymptomatic

ACI: Acute COVID-19 illness
OSC28: Ongoing symptomatic COVID-19 (28-83 days)
PCS84: Post COVID-19 syndrome (≥84 days)
NC28: Non-COVID-19 illness 28-83 days
NC84: Non-COVID-19 illness ≥84 days

ApoA1: Apolipoprotein A1
ApoB: Apolipoprotein B

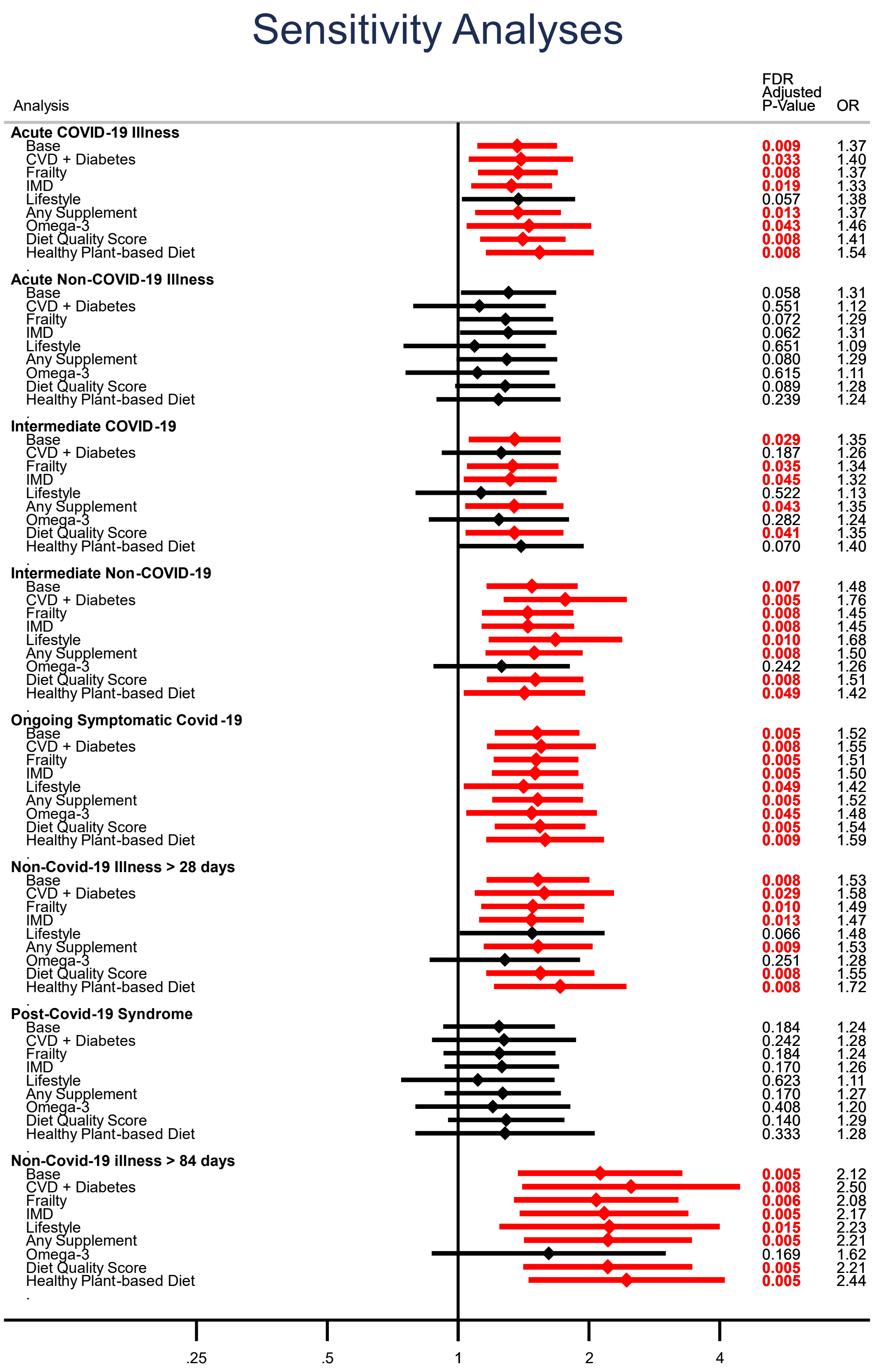

Supplementary Figure 2: Sensitivity analyses comparing the effect of additional variables on the risk of illness phenotype, compared to asymptomatic Covid-19.

Base is our model adjusted for age, sex and body mass index.

95% Confidence intervals displayed with p-values adjusted using Benajmini-Hochberg False Discovery Rate correction.

Red indicates FDR corrected p-value <=0.05

CVD – Self-reported cardiovascular/Heart disease; IMD – Index of Multiple Deprivation;

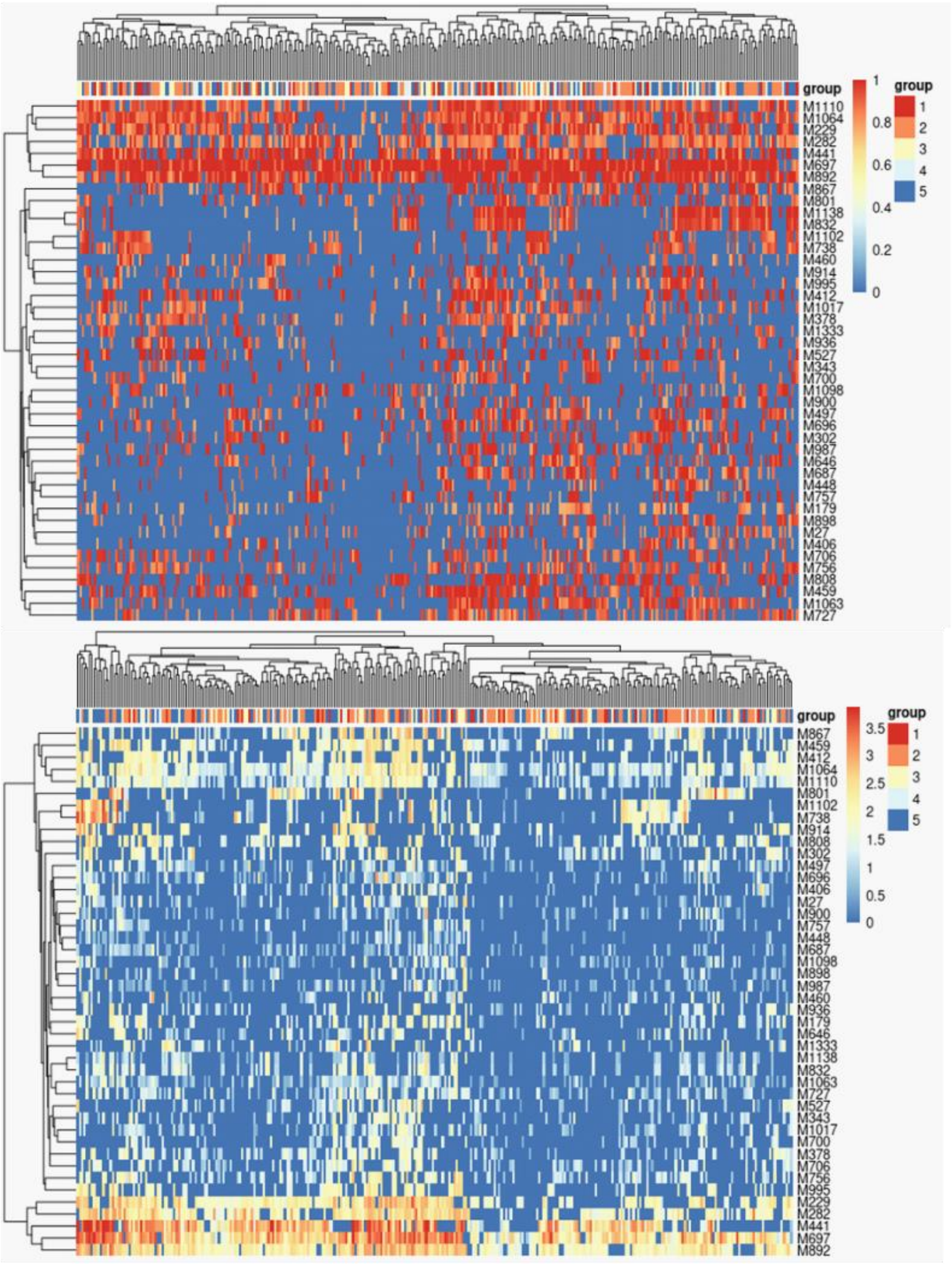

Supplementary Figure 3: Quality control of A) Breadth of coverage and B) Depth of Coverage for microbiome sequencing cohort. Sample (x-axis) and markers (Y-axis) are coloured based on breadth of coverage (ensures marker genes are sequenced) and depth of coverage (determines depth of sequencing)

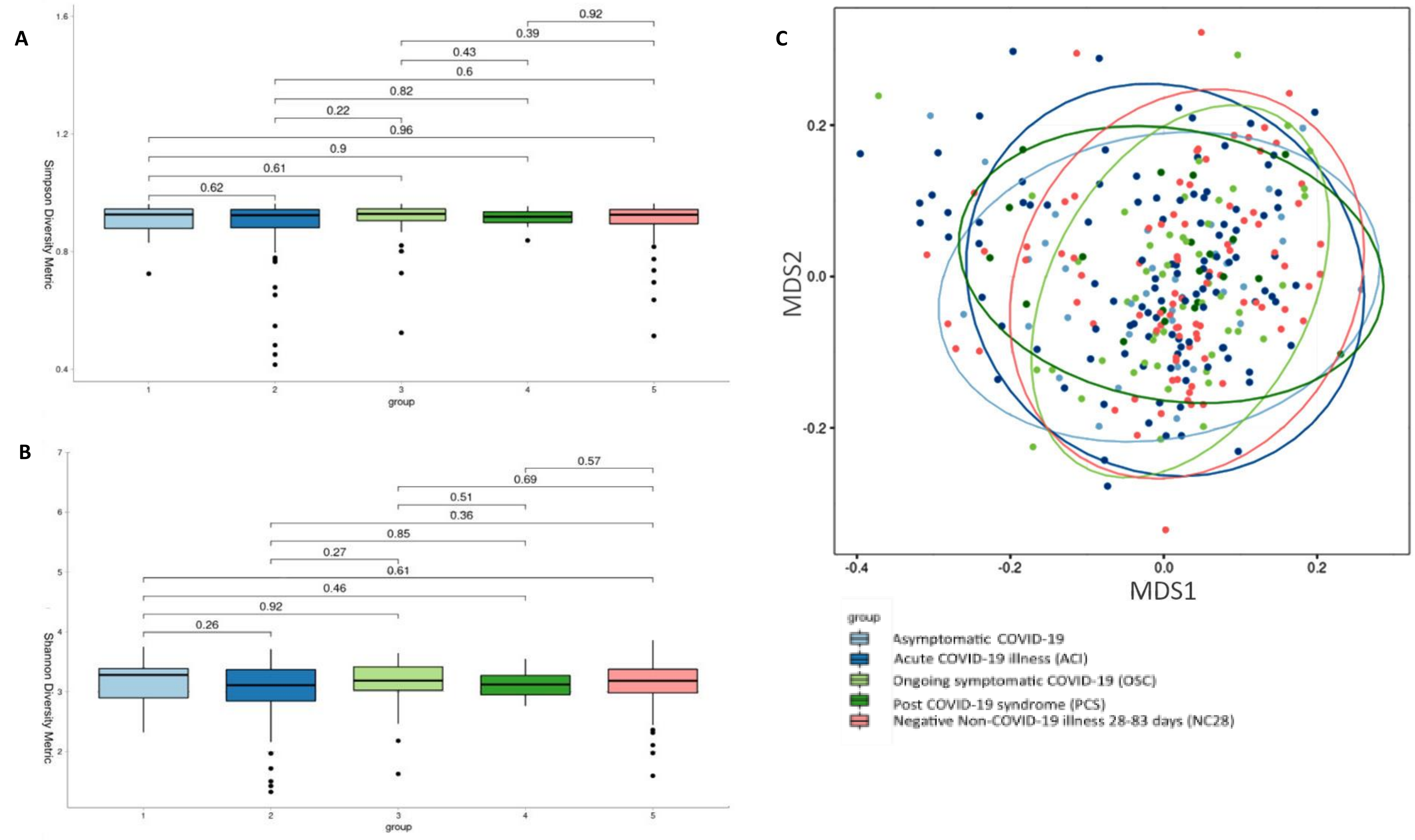

Supplementary Figure 4:
A) Simpson alpha-diversity and (B) Shannon alpha-diversity
(C) Metric Multidimensional Scaling (MDS) based on Bray Curtis dissimilarity metrics, showing the dissimilarity between five groups. MDS ordination based on Bray-Curtis dissimilarity was used to visualize the dispersion of microbial community among groups. The variance explained by the first dimension is 5.1%, and for the second dimension 3.1%.
Ellipses represent 95% Confidence Intervals.

Supplementary Figure 5: Graphical overview

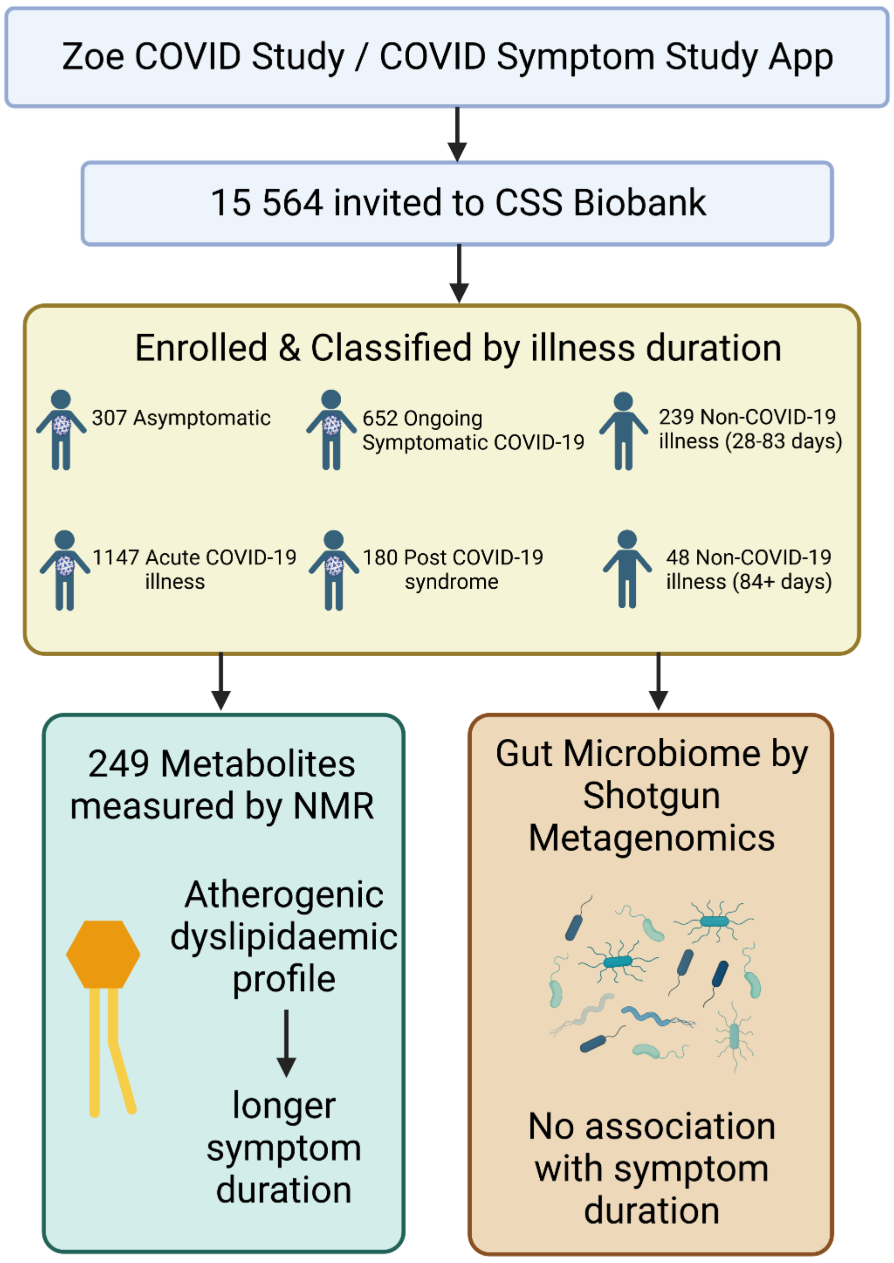

Supplement: Supplementary file 2 — Supplementary Information 2. [file 41598_2023_34598_MOESM2_ESM.pdf]
